# A Mechanism for the Polarity Formation of Chemoreceptors at the Growth Cone Membrane for Gradient Amplification during Directional Sensing

**DOI:** 10.1371/journal.pone.0009243

**Published:** 2010-02-22

**Authors:** Cedric Bouzigues, David Holcman, Maxime Dahan

**Affiliations:** 1 Laboratoire Optique et Biosciences, Inserm U696, CNRS 7645, Ecole Polytechnique, Palaiseau, France; 2 Département de Biologie, Ecole Normale Supérieure, Paris, France; 3 Laboratoire Kastler Brossel, CNRS UMR 8552, Ecole Normale Supérieure, Université Pierre et Marie Curie-Paris 6, Paris, France; University of Oldenburg, Germany

## Abstract

Accurate response to external directional signals is essential for many physiological functions such as chemotaxis or axonal guidance. It relies on the detection and amplification of gradients of chemical cues, which, in eukaryotic cells, involves the asymmetric relocalization of signaling molecules. How molecular events coordinate to induce a polarity at the cell level remains however poorly understood, particularly for nerve chemotaxis. Here, we propose a model, inspired by single-molecule experiments, for the membrane dynamics of GABA chemoreceptors in nerve growth cones (GCs) during directional sensing. In our model, transient interactions between the receptors and the microtubules, coupled to GABA-induced signaling, provide a positive-feedback loop that leads to redistribution of the receptors towards the gradient source. Using numerical simulations with parameters derived from experiments, we find that the kinetics of polarization and the steady-state polarized distribution of GABA receptors are in remarkable agreement with experimental observations. Furthermore, we make predictions on the properties of the GC seen as a sensing, amplification and filtering module. In particular, the growth cone acts as a low-pass filter with a time constant ∼10 minutes determined by the Brownian diffusion of chemoreceptors in the membrane. This filtering makes the gradient amplification resistent to rapid fluctuations of the external signals, a beneficial feature to enhance the accuracy of neuronal wiring. Since the model is based on minimal assumptions on the receptor/cytoskeleton interactions, its validity extends to polarity formation beyond the case of GABA gradient sensing. Altogether, it constitutes an original positive-feedback mechanism by which cells can dynamically adapt their internal organization to external signals.

## Introduction

During the development of the nervous system, neurons navigate to find their correct targets and to form a functional nervous network [1], [2]. Growing axons modulate their elongation direction in response to asymmetric distributions of attractive or repulsive diffusible chemical signals, such as neurotrophins [3], [4], netrins [5], semaphorins [6], homeoproteins [7] or neurotransmitters [8], [9]. The detection of guidance cues occurs at the mobile end tip of the axon, the growth cone (GC), which acts as a chemical sensor. Asymmetric activation of membrane receptors triggers the oriented remodeling of the cytsokeleton and subsequent attractive or repulsive steering of the GC [10]. A remarkable feature of GCs is their ability to sense concentration differences across their cellular extent below a couple of percents [11], [12]. Accurate responses to a directional signal have also been reported during chemotaxis in amoebas or neutrophils [11], [13], [14], [15]. In these eukaryotic cells, chemotaxis involves an asymmetric reorganization or compartmentalization of signalling molecules within the cell [16], [17], [18], [19]. The formation of such a cell polarity presumably serves for signal amplification, by turning a weak external gradient into a steeper internal one.

Compared to amoebas or neutrophils, the gradient-induced dynamic reorganization within a GC during axonal guidance has been less investigated, possibly because of the multiplicity and complexity of the signaling pathways. Nevertheless, several studies have pointed to major spatial rearrangements and polarized signaling processes in the GC response. The asymmetric localization of actin-mRNAs have been reported, suggesting that GC steering follows a local and polarized translation [20], [21]. Similarly, in the presence of a BDNF (Brain-Derived Neurotrophic Factor) gradient, membrane receptors preferentially associated to lipid rafts localized on the side of the GC facing the gradient source [22], possibly causing a modulation of the cell response [23]. However, the mechanisms by which molecules (proteins, mRNAs,…) or organelles are asymmetrically translocated remain unclear.

**Figure 1:**
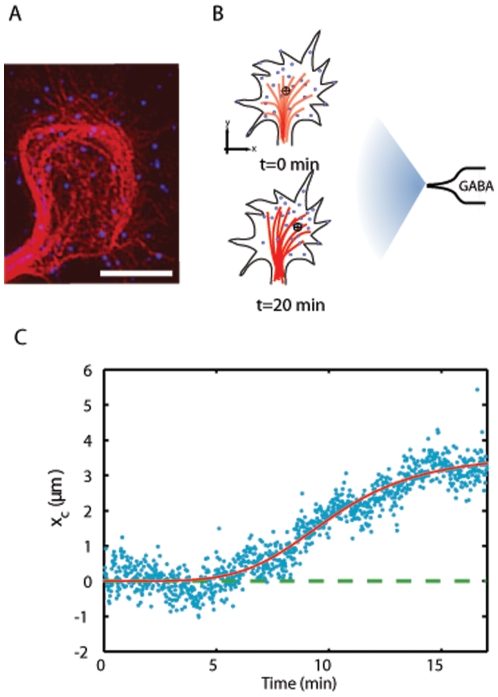
Experimental results. **A**. Axonal growth cone with microtubule staining (red) and QD labeling of γ2 sub-unit of GABA_A_Rs (blue) in the absence of stimulation. The scale bar represents 10 µm. **B**. Model for the redistribution of the GABA_A_Rs (blue dots) and MTs reorganization (red lines) in a GC membrane submitted to a GABA gradient (grey arrow). A pipette is placed perpendicularly to the axon axis at ≈100 µm of the GC and ejects GABA periodically (2 Hz) to create a permanent gradient. The average position of the receptors is marked by the black target. **C**. Time-evolution of the average GABA_A_Rs position 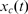 along the x-axis [24]. The red curve is a heuristic fit 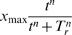 providing the redistribution half-time 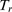 = 10.3+/−0.2 min, amplitude 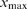 = 3.6+/−0.1 µm and 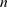 = 4.9+/−0.4.

Recently, our group has investigated the membrane organization of GABA receptors in the GC of spinal cord neurons during GABA gradient sensing using a single molecule assay [24]. Studies had shown that GABA and other neurotransmitters such as glutamate or acetylcholine, can mediate GC attraction by modifying the MT organization [8], [9], [24]. We reported that prior to GC steering, a GABA gradient induces a microtubule (MT)-dependent receptor redistribution towards the source of GABA (Figure 1A). Moreover, during the polarity formation at the GC membrane, the intracellular calcium, a secondary messenger in GABA-induced signaling [8], [25], showed an increase in the asymmetry of its concentration [24]. Altogether, these observations suggested that, during the phase of directional sensing that precedes cell steering and motility [15], the polarized redistribution of chemoreceptors serves as an amplification process in gradient sensing.

Several questions remain concerning the formation and maintenance of a polarized distribution of chemoreceptors: by which mechanisms do receptors become asymmetrically localized? Which physical or biochemical process primarily determines the time-scale of the redistribution? How does the redistribution and amplification depend on the characteristics of the external concentration profile (mean concentration and slope)? Addressing these questions and their physiological implications during axonal guidance requires a quantitative description of the receptor spatiotemporal organization within the GC membrane.

Here, we construct a computational model to investigate the physical and biochemical processes that govern the distribution of the receptors. We describe the receptor dynamics by a combination of lateral diffusion and MT-dependent transport. In addition, we introduce a coupling between the activity of the receptors in the external gradient and the MT dynamics. All the parameters in the model are derived from experiments. With this approach, we are able to reproduce all our experimental observations on the formation and maintenance of polarity at the growth cone membrane. We also make predictions on the operating properties of GCs as a sensing, amplification and filtering module. Our results emphasize the role of diffusion in the emergence of a spatial organization and suggest that the kinetics of the polarity formation is determined by Brownian motion rather than by specific interactions. Importantly, the results presented below are largely independent of the details of the molecular interactions and of the kinetics of the biochemical signaling reactions. As a result, our model has a validity that extends beyond the specific case of GABA_A_ gradient sensing and provides a general framework for the study of cell polarity [26], [27].

## Results

### Experimental Results

We first summarize the experimental results that have served as a basis for our modeling effort. We recently introduced a single-molecule assay in which individual GABA_A_ receptors (GABA_A_Rs), tagged with quantum dots (QDs), are tracked in the GC of cultured spinal cord neurons over extended periods (up to 30 minutes) [24]. In the presence of a GABA gradient released by a pipette positioned perpendicularly to the axon axis, the receptors asymmetrically redistributed accross the GC towards the source of GABA (Figure 1A). The redistribution occured in 10–20 minutes, prior to GC steering, and was completely reversible when switching off the gradient. On this time scale, no endocytosis of the receptors was observed and all the tagged-receptors remained in the GC membrane [24]. Furthermore, the spatial rearrangement of GABA_A_Rs could be abolished by using gabazine, a specific antagonist of GABA_A_Rs, or by depolymerizing MTs using nocodazole [24]. Concomitantly to the establishment of polarity within the GC membrane, we measured an enhancement in the asymmetry of intracellular concentration of calcium [24], suggesting that the formation of a polarized distribution of GABA_A_Rs serves as an amplification step in gradient sensing.

A quantitative measurement of the dynamics of polarity is obtained by computing the average position 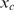 of the tagged GABA_A_Rs along the gradient axis 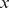 and perpendicular to the axon axis (Figure 1). The time evolution of 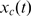 can be separated in three stages: (i) an initial latency period (for 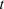
<5 mn) during which the distribution remains symmetric with respect to the 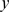-axis, (ii) an intermediate redistribution phase (for 5 min<
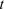
<15 min) during which the distribution shifts towards the GABA source, (iii) a final steady-state (for 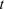
>15 mn) when 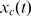 reaches a saturating value 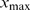. We fitted 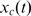 using the phenomenological law 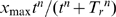 (Figure 1B) and derived the redistribution half-time 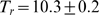 min and the exponent 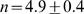. We further analyzed with single-molecule tracking experiments the mechanisms involved in the establishment of a polarized distribution. In the presence of the external gradient, the receptors did not appear to immobilize at asymmetrically located anchoring points and, instead, constantly moved in the cell membrane. This excludes a conventional “diffusion-trap mechanism”. Since sole diffusion can not lead to a polarized distribution, GABA_A_ receptors have to undergo active transport, presumably due to interactions with the cytoskeleton. Using cytoskeleton-depolymerizing agents and advanced methods for the analysis of single-molecule trajectories [28], we determined that receptors had a conveyor-belt motion in which they alternate between free diffusion and MT-dependent directed movement. The diffusion coefficient 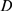 of GABA_A_Rs in GCs was 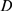 = 0.25 µm^2^.s^−1^ [24], characteristic of a protein freely diffusing in a cell membrane [29]. The average interaction time between GABA_A_Rs and the MTs was ∼4 s and was not modulated by receptor activity. The suppression of MT-oriented movement by a taxol treatment blocking MTs in their polymerized state, favors the hypothesis that receptor movements resulted from MT polymerization, possibly through direct or indirect interactions of the GABA_A_Rs with MT ends.

Based on these experimental results, we proposed a simple qualitative model of positive feedback between GABA-induced signaling and dynamics of the MTs to describe the spatiotemporal dynamics of GABA_A_Rs [24]. In brief, activation of the receptors induces remodeling of the cytoskeleton with preferential growth of the MTs towards the GC leading edge. In turn, this oriented elongation causes a redistribution of the GABA_A_Rs toward the gradient source, resulting in an enhanced asymmetry in intracellular calcium and in amplification in gradient sensing. This sequence of events (detection, reinforcement and propagation of the spatial cue) is a common feature for the formation of polarity in many cellular systems [15], [30], [31]. In our case, however, a quantitative description of how the functional organization of the cell (the receptor polarized redistribution) arises from molecular properties (the receptors diffusion and their interactions with MTs) is yet to be obtained and is the subject of the following modeling effort.

### Model for the Coupled Dynamics of GABA_A_Rs and MTs

We present a mathematical model of GABA_A_Rs spatial organization based on the coupled dynamics of membrane receptors and microtubules. As shown below, this model reproduce prior experimental findings on the polarized distribution of the receptors in an external GABA gradient. Furthermore, it allows a predictive analysis of the cell response to gradient conditions that are yet to be experimentally investigated. While little is known about the interactions between GABA_A_Rs and MTs or the GABA-induced signaling pathway, the motion of the receptors has been precisely described with single QD measurements [28]. Consequently, our knowledge of GABA_A_Rs lateral dynamics serves as the main ingredient in our modeling approach.

**Figure 2:**
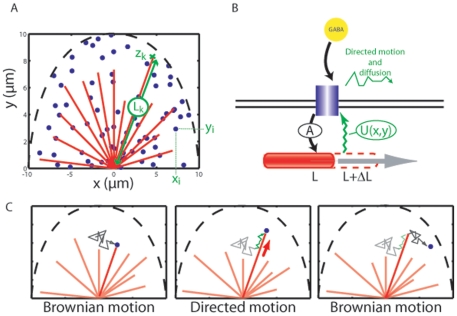
Model description. **A**. Geometry of the model : receptors are represented by blue dots, MTs by red lines and the limit of the GC is indicated by the dashed black line. **B**. Interactions between GABA receptors and MTs. Activated receptors stimulate MT growth (activation field 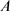, black arrow), which in turn alters the receptor dynamics (attractive interaction 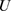, green line). **C**. Dynamics of a receptor. As a diffusing receptor encounters a MT (left), it undergoes a transient phase of transport due its interaction with the MT end (center). Eventually, it escapes the interaction potential and resumes its diffusive motion (right).

The GC is described as a bidimensional system with the shape of a 10 µm radius half-disk containing a constant number of 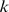 MTs and of 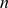 identical independant receptors. All MTs originate from the center of the half-disk and are modeled as stiff lines with fixed orientation (regularly distributed between 0 and 180°) and variable length 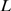 inferior to the GC radius (Fig. 2A). Before stimulation by the external gradient, the initial MT length 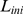 is 7 µm. These approximations are consistent with the GC geometry [32] and respects the GC symetries but exclude any GC steering or elongation in the simulation. In all this work, we focus on the phase of directional sensing that precedes GC turning.

Our hypothesis is that the receptor redistribution results from the combined effects of the membrane dynamics of GABA_A_Rs, of the elongation dynamics of MTs, and of their coupling induced by the receptor signaling activity [24]. We model these reciprocal interactions between MTs and GABA_A_Rs by: (i) a local attractive potential 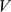 at the end tip of each MT, in which receptors can be transiently trapped, (ii) an activation field 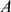 reflecting the instantaneous activity of the receptors in the GABA gradient, and (iii) a positive coupling between the MT length 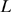 and the local field 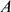. The detailed expression of 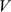 and 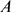 is given in the Materials and methods section and we only summarize here their main properties.

The potential 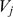 reflects the ability of receptors to interact with the 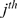 MT and is centered on the MT end tip. When summed over all the MTs, it leads to a time-dependent potential 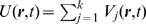, where 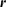 denotes the position in the xy plane. As a result, the lateral dynamics of the receptors within the GC is determined by their diffusion (with a coefficient 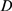) in the potential energy landscape 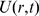 created by the MTs (see Materials and methods).

The stochastic dynamics of MTs has been widely investigated over the past decade [33], [34], [35]. It is governed by an alternation of depolymerization (catastrophes) and of polymerization events (rescues). In a GC, a complete description of the MT dynamics also requires a proper modeling of the signaling pathways coupling the MT remodeling to the receptor activity. We do not attempt at including such level of complexity in our model and, rather, propose a simplified approach describing the average behavior of a single MT sufficient to account for the regulation of its dynamics upon receptor activation. In practice, the dynamics of each single MT is given by the time-evolution of its length 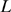, or equivalently by the position of its end tip. In the absence of coupling with the receptors, 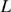 fluctuates around a steady state value 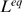 with a relaxation time 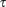 and a diffusion coefficient 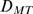 (see Materials and methods). When the GC is submitted to a GABA concentration gradient 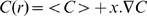 along the *x*-axis, the receptor activity, monitored by the value of the activation field 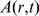, causes a modification of the MT equilibrium length 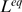 (Fig. 2). If the local activation 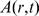 is higher than its average, the MT growth is locally favored and it is disadvantaged when it is lower. This can be captured by assuming that: (i) the activation field 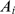 due to the 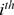 receptor located in 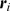 is proportional to the GABA concentration 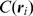, (ii) 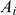 is centered on 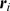, (iii) the total activation 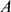 is the sum of all individual fields 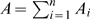 and (iv) the growth rate of 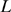 is proportional to the relative value of the local activation field 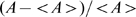 (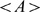 denotes the average value of 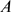 over the GC, see Materials and methods). The hypothesis of a linear relation between the activation field and the local GABA concentration has been chosen for simplicity. It is only valid when the external concentration is far from saturation and does not describe adaptation mechanisms to the value of the average concentration of guidance cue. However, such adaptation mechanisms, found for BDNF or netrin induced guidance [36], has not yet been reported for GABA signaling. Note also that our description does not account for molecular noise in the ligand binding, a process susceptible to contribute to the chemotactic GC response [37] but which was not required in our case to describe the receptors polarization.

The comparison of 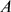 to its average value ensures that a uniform bath of GABA has no effect on the GC morphology, consistently with experimental observations [24]. This supposes the existence of a global variable acting at the whole GC scale, which can rely on a the action of a fast diffusing second messenger, such as Ca^2+^ ions allowing a local knowledge of the average GC response. We also tested the possibility that the growth rate depends on the comparison of 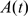 to a fixed value (its value at time 0), rather than to its instantaneous average, and it yielded comparable results for the receptor distribution (Figure S3). It is noteworthy that a similar form of adaptation has been also reported in chemotactic measurements on Dictyostellium amoeba which stably polarize in oriented signals but only transiently and in random directions when placed in a uniform stimulation [38].

The values of 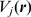 and 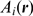 reflect the molecular interactions and biochemical reactions occuring in the signaling pathways connecting GABA_A_Rs to MTs. Since many molecular details on this pathway are lacking, we made the two following hypotheses to obtain a generic form. First, the response was assumed to be *local*, meaning that the spatial extension of 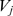 and 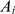 is small compared to the GC size. Second, the response was *instantaneous*, meaning that the time scale of the biochemical reactions was shorter than the time scale of the spatial dynamics. Based on these simplifying conditions (see Materials and methods), we have built a model which is robust to assumptions on the exact nature of the molecular interactions and thereby captures the dynamics of the GC reorganization without a detailed knowledge of the transduction biochemical pathway.

### Formation of a Polarized Distribution of Receptors

We first performed simulations with a set of paramaters consistent with experimental data to analyze the formation of a polarized distribution of receptors upon application of a gradient. We computed the dynamics of receptors in a GC submitted to a gradient of 10% (*i.e* with a difference of concentration of 10% between the two extremities of the GC) over a duration of 1000 s (see Materials and methods). The gradient axis was oriented perpendicularly to the GC axis. We assumed that the GC contained 200 randomly distributed receptors and 50 MTs. These values, which are further discussed below, are close to the density of receptors in the extra-synaptic membrane [39] and of MT in GCs [32] respectively. All the other parameters used in our simulations were chosen based on experimental data (see Materials and methods).

**Figure 3:**
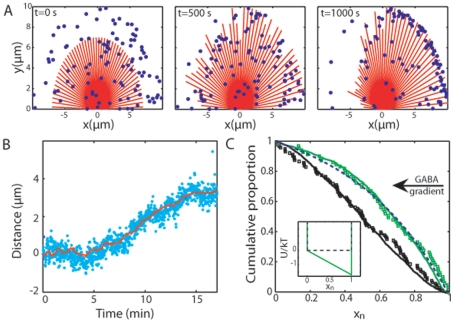
Results of the simulations. **A**. Distribution of receptors (blue dots) and MTs (red lines), successively at t = 0, 500 and 1000 s. **B**. Average redistribution of the GABA_A_Rs in numerical simulations (10 runs, red line) compared to experimental data (9 GCs, blue dots [24]). The amplitude 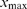 of the numerical redistribution is normalized to be the same as observed in experiments. **C**. Cumulative distribution of receptors 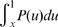 in experiments (squares) and in numerical simulations (plain line) before (black) and after (green) 1000 s stimulation. The dashed blue line is a fit by a Boltzmann distribution in an effective linear potential 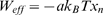 (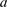 = 1.7). Inset: a plot of the effective potential before (black dashed line) and after (green plain line) the simulation.

The results of the simulation show a progressive redistribution of the receptors towards the proximal region of the GC as well as a remodeling of the MTs (Figure 3A). Similarly to the experimental data, the evolution of the simulated distribution of receptors was analyzed by plotting as a function of time the position 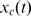 of the center of mass of the receptors along the gradient axis (Figure 3B). The curve 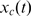 - obtained by averaging 10 runs of simulations - is in excellent agreement with the experimental results (Figure 3B). The amplitude 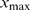 of the redistribution is comparable (respectively 14% and 15% of the GC width) between experiments and simulations. The receptors are displaced toward the source of GABA after similar typical lag times of 10 min in experiments and in simulations. Using the phenomenological fit 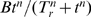 (where 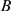, 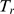 and 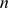 are free parameters), we determined 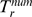 = 10.2±0.1 min and 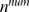 = 4.3±0.2, close to the experimental values 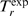 = 10.3±0.2 min and 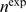 = 4.9±0.4. Therefore, numerical simulations successfully capture the dynamics of the MT-mediated organization of GABA_A_Rs in the GC, meaning that the interplay between receptors and MTs is sufficient to cause the polarization at the cellular scale.

### Steady-State Polarized Distribution of Receptors

Our model describes not only the time evolution of the average position 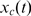 (see above) but also the receptor distribution in the final polarized steady state. Indeed, we have determined, experimentally and numerically, the complete distribution of receptors 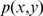 in the GC membrane, before and after stimulation by a GABA gradient for 1000 s. Initially, the distribution 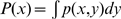 along the gradient axis was symmetric with respect to the GC axis. After stimulation, the distribution 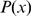 is biased toward the source of the gradient (Fig. 3C), with about two-third of the receptors in the region of the GC facing the gradient source.

The distribution 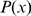 is correctly approximated using the equilibrium thermodynamical distribution 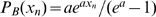 in a one-dimensional linear potential 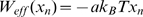 (where 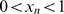 is the position normalized by the GC size and 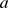 is a dimensionless parameter describing the depth of the effective potential 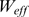 in 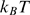 units. In other words, the distribution of receptors corresponds to the equilibrium distribution for an effective potential proportionnnal to the external GABA gradient 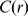. When fitting the experimental and the simulated distributions, the values of 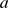 are identical: 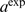 = 1.7±0.1 and 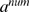 = 1.73±0.01 respectively. This corresponds to a receptor concentration ∼5 times higher at the front edge (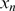 = 1) than at the trailing edge (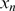 = 0).

### Sensitivity of the Formation of Polarity to Parameter Values

We tested the sensitivity of the numerical results to parameters such as the number 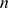 of receptors, the number 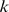 of MTs and the diffusion coefficient 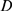. Unless otherwise mentioned, the values used in the simulation were 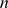 = 200, 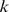 = 50 and 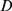 = 0.25 µm^2^.s^−1^. The values of all the other parameters, kept constant in the simulations, are indicated in Table 1 and a complete summary of the dependence of 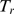 and 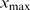 on the different experimental and modeling parameters is given in Table 2.

**Table 1:** Summary of the different parameters used in the simulations.

Parameter	Physical meaning	Value	Source
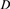	Diffusion coefficient of the receptors	0.25–2 µm^2^.s^−1^	Measured [28]
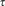	Relaxation time of MT elongation	10 s	Inferred from MT speed measurement [59]
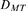	Effective diffusion coefficient of the MT length	0.01 µm^2^.s^−1^	Inferred from MT speed measurement [59]
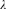	Influence of receptor activation on MT dynamics	0.1 µm.s^−1^	Inferred from MT speed measurement [59]
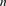	Number of receptors	10–10000	Tested in the simulation
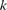	Number of MTs	10–200	Measured [32] and tested in the simulation

**Table 2:** Effects of the increase of different parameters on the receptor redistribution half-time and amplitude: − indicates a decrease, + an increase, and ∅ the absence of effect.

	Half-time 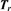	Amplitude 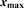
Number 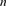 of receptors	∅	++
Number 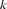 of microtubules	−	∅
Diffusion coefficient 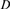	−−	−−
Gradient 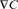	∅	++

**Figure 4:**
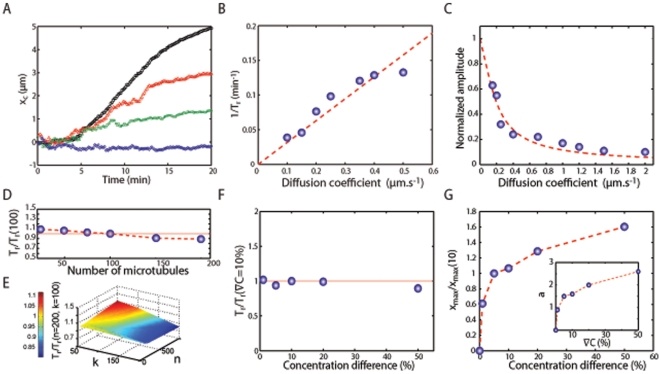
Exploration of the parameters. **A**. Time evolution of the average position of the receptors along the gradient axis for different numbers of receptors: 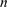 = 10 (blue diamonds), 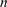 = 50 (green squares), 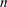 = 100 (red circles) and 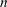 = 10000 (black triangles) **B–C**. Inverse of redistribution time 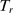 (B) and amplitude of redistribution (C) normalized by the GC size as a function of the diffusion coefficient 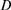. The red dashed line on (B) is a linear fit. The results (circles) in C have been adjusted by the variation with temperature expected for a Boltzmann distribution in a linear potential 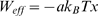 (dashed curve)). **D**. Redistribution time 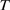 for different numbers of MTs 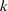 (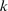 = 10, 75, 100, 150, 200) normalized by the value 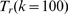 obtained for 100 MTs. **E**. Redistribution for different number of receptors (n) and MTs (k) normalized by the redistribution time obtained for 100 MTs and 200 receptors. **F**. Redistribution time 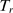 as function of the gradient 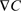. All times are normalized by the redistribution time 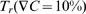 for 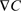 = 10%. **G**. Relative amplitude 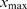 of the redistribution normalized by 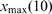 as a function of 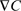. Inset: value of the corresponding effective potential depth 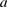 at equilibrium.

First, we analyzed the role of the number of receptors. Since the density of GABA receptors is not precisely determined in GCs, we initially hypothesized that it compares to the one of free GABA_A_Rs in rat cerebellar granule cells, which is ≈2–3 µm^−2^ [39]. Such a density would result in a total number of a few hundreds of receptors in the GC. Consequently, we have performed numerical simulations with a number 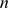 of GABA_A_Rs ranging between 10 and 10,000 (Figure 4A and Table 2). We found that the redistribution occurred for any 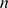
>10. The half-time 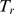 remained approximately 10 min (Fig. 4E) for all values of 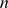, while the redistribution amplitude 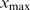 increased with the number of receptors (Fig 4A). The latter result was qualitatively expected since the number 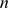 of receptors regulates the strength of the coupling between MT and receptor dynamics and, therefore, the value of 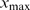.

Next, we tested the role of the number 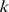 of MTs. Simulations showed that the half-time 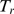 slightly decreases for increasing values of 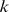 (Figure 4D and 4E). However, the value of 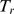 did not change by more than 10%, proving an overall robustness of the model with respect to the exact properties of the MT network. We also computed the amplitude 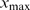 as a function of 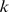 and found that 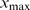 was largely insensitive to the number of MTs. Altogether, the results of the simulations indicate that the time required for the formation of polarity at the GC membrane is not due to a particular adjustment of the input parameters (Fig. 4E). Rather, it emerges as a constitutive property of the model that can be obtained for a wide range of physiologically relevant parameters.

As shown above, the dynamics of the receptors and of the redistribution is controlled to a large extent by diffusion. We performed simulations for values of 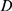 comprised between 0.1 and 2 µm^2^.s^−1^. In all cases, the redistribution of receptors in the GC membrane occured. However, the redistribution half-time 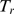 decreased with increasing diffusion coefficients. More precisely, the variation of 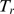 was correctly approximated by an inverse law 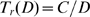 (Figure 4B and Table 1). Such dependence is characteristic of the time needed for a particle, diffusing with coefficient 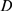 in a two-dimensional domain, to find a target of finite size [40]. This observation, which is further discussed below, suggests that Brownian diffusion plays a key role in the latency period, prior to receptor redistribution.

The amplitude 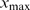 also depended on the diffusion coefficient and decreased with increasing values of 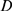. This can be understood by considering that a variation of 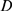 at equilibrium is equivalent to a variation of the temperature 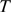. The increase of 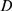 makes receptors less sensitive to the effective potential due to the interactions with the MTs and thus impairs the receptor redistribution. The variation of the redistribution amplitude with 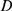 is moreover consistent with the presence of a linear potential due to the MT reorganization (Fig. 4C). Altogether, the results of the numerical simulations illustrate a double role for the diffusion. On the one hand, a high value of the diffusion coefficient 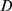 accelerates the redistribution by reducing the half-time 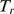, but, on the other hand, it increases the fluctuations and, as a result, diminishes the degree of polarization.

### Dependence of the Polarity on the External Gradient

Numerical simulations were also used to determine the effect of the gradient parameters on the formation of polarity. In our model, the activation field 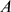 is proportional to the concentration gradient and its effect on the MT dynamics (through a modification of the MT equilibrium length) depends on the local value of 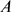 (see Materials and methods). This means that the average GABA concentration 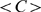 does not play a role and that the receptor redistribution solely depends on the concentration relative slope 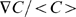. In the following, 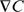 is expressed in units of the relative concentration difference between the leading and the trailing edge of the GC.

We performed simulations for gradient varying between 2% and 50% and, in all cases, a polarized distribution of receptors was obtained. However, changing the slope has different effects on 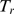 and 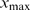. It appeared that 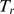 was independent of the slope (Figure 4F). Therefore, the kinetics of the redistribution is not determined by the activity-induced growth dynamics of MTs but, rather, is limited by the diffusion of receptors. In contrast, the redistribution amplitude 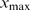 increases with 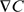 (Figure 4G). For all values of 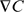, the distribution 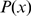 was described by an thermodynamical equilibrium distribution in a linear potential 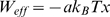 (Figure S1). However, the parameter 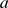 was not proportional to 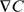 but exhibits a saturating behavior (inset in Figure 4G). This result is qualitatively expected considering the geometrical constraints of our model. Indeed, MTs can only extend up to a finite length, therefore putting a limit on the asymmetry of the effective potential determined by the interactions at the MT ends.

## Discussion

### Mechanism of Formation of Polarity

We have developed a model able to reproduce the asymmetric relocalization of membrane GABA_A_ receptors during GABA gradient sensing. The polarized self-organization of membrane receptors results from the coupling between receptor diffusion, receptor activation and the elongation dynamics of MTs. The coupling creates a positive feedback loop that mutally reinforce the receptor asymmetric localization and the MT oriented remodelling, resulting in a polarization of the cell membrane. With a set of parameters consistent with available data on GCs (number of receptors and MTs, membrane diffusion coefficient,…), we account *in silico* for both the redistribution kinetics and the polarized steady-state distribution of receptors in living neurons. Importantly, our model involves only minimal assumptions on the biochemical nature of the interactions between the receptors and the MTs.

In the model, the polarization depends on the collective behavior of a large number of membrane receptors and MTs. Unfortunately, an equation for the temporal evolution of the receptor distribution 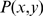 can not be simply derived. Nonetheless, the dependence of the redistribution on the different simulation parameters (diffusion coefficient, gradient) suggests a simple picture that captures the main elements in the formation of polarity at the membrane. First, during the latency period (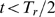), there is no asymmetry in the receptor distribution. The duration of this phase can be viewed as the time needed for diffusing receptors to reach their targets, i.e. the MT end tips in a region of strong activation. Accordingly, this duration is diffusion-limited and does not depend on the characteristics of the external gradient (Figure 4F). After this initial period, receptors reach a second phase in which they are transported by MTs towards the gradient source. In turn, the receptor asymmetric localization reinforces the asymmetric MT growth, providing a positive feedback. Finally, the distribution of receptors reaches an asymmetric steady state. In this last phase, the polarized distribution results from a competition between oriented transport, which reinforces the asymmetry, and lateral diffusion, a recycling process which tends to restore homogeneity.

These three phases can be viewed as a succession of exploration, transport and equilibrium. Their identification provides an original picture for the formation of polarity at the cell membrane in which the reorganization dynamics is dominated by the lateral motion rather than by the biochemical properties of the transduction pathway. It differs from the most common view of polarity, in which the role of the cytoskeleton is restricted either to the maintenance of the polarized state [14], [17] or to the motility subsequent to gradient sensing [41]. Our model is not limited to the particular question of GABA receptor organization but, rather, constitutes a generic approach to understand how spatial order within the cell can arise due to reciprocal coupling between signalling elements (here the receptors) and transport structures (the cytoskeleton). It can be compared to models introduced to describe the self-polarization of yeast cells, in which localized patches of activated cdc42 can spontaneously form [14], [42], [43] and result from membrane-cytoplasmic exchanges [44]. In particular, it was shown that the maintenance of this polarity can be explained as an interplay between diffusion, actin-based transport and endocytosis of cdc42 [14]. This description is conceptually close to ours, although it involves additional endocytotic recycling processes.

### Amplification and Temporal Filtering in Gradient Sensing

An important aspect of the dynamic relocalization of chemoreceptors is its relation to amplification and temporal filtering during gradient sensing. By bringing more signalling molecules on the leading side of the cell, a shallow external gradient can be potentially turned into a steeper internal one. In our experiments, we indeed measured an asymmetry of receptor concentration with a factor ∼5 between the back and leading edges in the presence of a 10% gradient. This was accompanied by an increased asymmetry in intracellular calcium concentrations, suggesting an amplification in the detection of GABA gradient [24].

Based on the study of the chemotactic response of amoebas and neutrophils, amplification in gradient sensing in eukaryotes is considered to rely on the combination of local excitation and global inhibition (LEGI) [11], [45], [46], [47], [48]. For instance, a second messenger (such as PI3K or PTEN) is activated rapidly and locally while its production is slowly and globally inhibited. Two-LEGI models for both PI3K and PTEN membrane binding sites have thus shown a good agreement with experimental results [45], [47]. Our results and model in GCs thus notably differs from previous observations in amoebas and neutrophils. First, in *Dictostelyum* amoebas, chemoreceptors remain uniformly distributed and the polarized cellular state is obtained by asymmetrically activating and localizing signalling lipids and proteins. This asymmetric localization is not cytoskeleton-dependent but, instead, results from a diffusion-trapping meachanism in the cytoplasm and at the cell cortex. Second, external gradients induce a switch-like response, which has been modeled as a phase separation within the cell [13].

In GCs, the distribution of chemoreceptors does not show a switch-like behavior and the degree of polarity, measured by the value of, varies with the value of. Our hypothesis is that such a modulation of the spatial distribution of receptors is an initial amplification step in the GC response to concentration gradient. Higher amplification could be subsequently achieved through non-linear properties of the biochemical signalling pathways. A recent computational study further supports the idea that spatial organization of membrane receptors plays a role in nerve chemotaxis [49]. By modeling the lateral dynamics of DCC receptors and the kinetics of the associated signaling pathway, Causin and Facchetti found that the receptors asymmetric relocalization was a key precursory event for chemotactic response to netrin gradients.

Temporal filtering in the GC response results from the fact that the relocalization of receptors occurs only after a duration 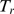. In other words, the GC acts as a low-pass filter and fluctuations in the concentration of guidance cues with frequency higher than 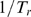 are averaged out. Furthermore, our simulations suggest that the cut-off frequency 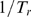 does not depend on the strength of the coupling between receptors and cytoskeleton but, instead, is determined by the diffusion coefficient 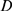 in the membrane (Fig. 4B). Experimentally and numerically, the value of 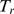 in GCs was found to be ∼10 minutes. This contrasts with the observations made in amoebas in which the polarization occurs much more rapidly, in a few seconds [50]. This discrepancy might simply reflect the different roles of gradient sensing in neural cells and amoebas. Indeed, the foremost physiological requirement of axonal guidance is its accuracy. A temporally averaged response to external signals might enable a more robust response and minimize navigation mistakes by focusing on persistent signals and by rejecting transient fluctuations in guidance cues. On the contrary, gradient sensing in amoebas is associated to hunting and food-searching. It might favor an almost instantaneous response, even at the risk of an increased error rate.

### The Role of Diffusion

In our model, the formation and maintenance of a polarized distribution of receptors results from an interplay between MT-induced transport and diffusion. Our results emphasize the important role played by Brownian motion, a process which neither requires specific molecular interactions nor consumes energy [51]. In our system, the value of 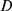 is essential to account for the polarization time-course and the amplification and temporal filtering in gradient sensing. As shown above, the polarization kinetics (determined by the value of 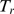) is diffusion-limited and the degree of polarization (measured by the parameter 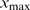) decreases rapidly with increasing values of 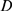 (Figure 4C). For a small 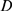, the redistribution time 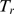 would be very large (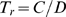), simply because receptors would take a long time to reach the MT ends. A sufficiently large diffusion coefficient is thus required to allow a receptor redistribution in a biologically compatible time. However, too large a diffusion coefficient prevents receptor relocalization (Fig. 4C) by making them insensitive to the effective potential created by the MTs. Diffusion is also important for the ability of the cell to respond to dynamic environments and to rearrange its membrane sensing machinery. In particular, receptors can diffuse back to a non-polarized distribution when the external gradient is switched off [24].

From a more general standpoint, tuning the diffusive properties could be a way to regulate the response of polarized or chemotactic cells to external signals. In fact, a modulation of the ratio of fast and slowly diffusing membrane receptors populations has been recently reported *in vivo* in amoebas [52]. This modulation was proposed to contribute to the asymmetric regulation of signal transduction and to amplification in gradient sensing. In neurons, a differential membrane mobility in the GC of pioneering and follower neurons [53] has been observed. Diffusion is slower in the pioneering GCs that serve as guide of following fasciculated neurons [54]. According to our model, pioneering neurons would respond more slowly and more reliably to external signals than follower neurons. The efficiency of the wiring in the nervous system would then be ensured by the sensitive response of pioneer neurons guiding the fast response of followers.

### Conclusion

In this work, we have developed a microscopic model to describe the establishment of a polarized response at the GC membrane during nerve chemotaxis. This model is a simple and generic approach that can explain the emergence of asymmetric cellular organization in many contexts. In the case of GABA gradient sensing, we explained, using numerical simulations without adjustment of the parameters, the polarization kinetics and the steady-state polarized distribution of GABA_A_ receptors. Our results support a new mechanism for the dynamic cellular reorganization during gradient sensing different from the commonly accepted one in the chemotactic response of amoebas and neutrophils.

We used our model not only to quantitatively reproduce prior experimental observations but also to make predictions about the sensing, amplification and filtering properties of GCs. In future experiments, one could directly check whether: (i) the redistribution time 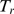 is independent of the gradient, (ii) the steady-state polarized distribution of receptors corresponds to the Boltzmann distribution in a potential proportional to the external concentration profile. To test the GC response to various external stimulations, it is necessary to precisely adjust the concentration profile of chemical cues. Conventional guidance assays based on the pulsative release by a pipette do not provide sufficient control of the profile. Other assays using gradient imprints in collagen gels are more accurate but do not have the temporal resolution required to probe the gradient-induced cellular polarization [12], [37]. However, the recent advent of microfluidic-based assays now permits gradients to be applied on cultured cells and open perspectives for quantitative investigations of the neuronal response to guidance signals [55], [56], [57].

## Materials and Methods

### Single Quantum Dot Imaging of GABA_A_Rs

An extensive discussion of experiments performed on living neurons can be found in references [28] and [24]. Cultured spinal neurons at 3–6 days in vitro were submitted to a GABA gradient using a conventional guidance assay with a pipette placed at 90° from the axis of the parent axon [24]. GABAR 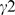 subunits, known to be present in functional receptors, were labeled with biotynilated antibodies and streptavidin-coated QDs. Time-lapse sequences of fluorescence images were acquired at 1 Hz to investigate the spatial organization of the chemoreceptors. In the presence of the gradient, QD-tagged receptors redistributed across the GC membrane towards the GABA source in ∼10–15 minutes (Figure 1A). On the time scale of our experiments (≤20 minutes), the morphology and orientation of GCs remained stationary, such that motion of receptors was due to their lateral dynamics in the membrane and not to a global translocation of the cell. In each image, we determined the position of the QDs with a ∼30 nm resolution by fitting the fluorescence spots with a 2D gaussian curve [58]. Determination of each QD fluorescence spot position provided the spatial distribution of receptors in the GC as a function of time [24].

### Numerical Computations

All the simulations were performed with custom algorithms written with Matlab (Mathworks, MA). Brownian motions in two dimensions 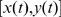 are simulated by the generation at each time 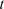 of two continuous Gaussian variables 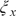 and 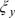 (with 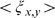 = 0 and 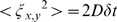, where 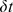 is the time increment and 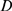 the diffusion coefficient) such that 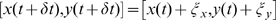. In our simulations, positions and quantities depending on the positions (such as 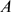 or 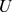) were actualized every 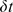 = 10 s. With this procedure, the calculation time of a single run of simulation (*i.e* 10 numerical experiments with the same set of parameters) was about 5 minutes with a standard computer (PC Pentium 4 3 GHz, 1Go RAM).

### Mathematical Model for the Receptor and MT Dynamics in a Concentration Gradient

The GC is placed in a concentration profile 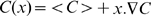 oriented along the x-axis, perpendicular to the parent axon.

We define the MT-receptor interaction potential 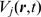 due to the 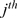 microtubule by:
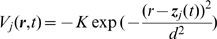
(1)where 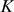 is a positive constant, 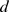 the extension of the interaction potential, 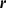 is the 2D spatial coordinate and 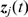 the position of the 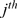 microtubule end. Therefore, the 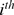 receptor (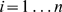) diffuses in the energy landscape 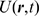 given by:
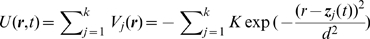
(2)and its motion is described by the Langevin equation:
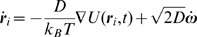
(3)where 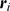 is the two-dimensional position of the 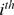 receptor, 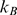 the Boltzmann constant, 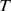 the temperature and 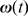 a two-dimensional Brownian variable.

We model the dynamics of MTs by:
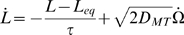
(4)where 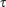 is the relaxation time of the MTs toward equilibrium 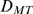 the effective diffusion coefficient of the MT length and 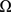 a Brownian variable.

For the 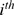 receptor, located at position 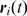, the activation field 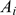 is proportional to the concentration 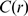 and is given by:
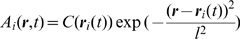
(5)where 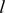 is the field extension. The total activation field 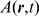 is obtained by summing up the contribution of all the individual receptors:
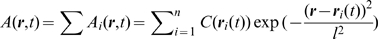
(6)The Gaussian shape for the fields 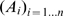 and the potentials 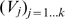 is discussed in the next paragraph.

Finally, we define the coupling between the receptor activity and the MT dynamics by considering that deviations of the activation field from its average lead to a modification of the equilibrium MT length. We introduce a relation between 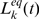, the equilibrium length of the 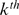 MT, and 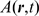. 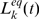 is a markovian variable with a growth rate given by:
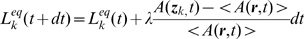
(7)Where 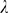 is a positive constant and 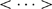 denotes spatial averaging over the entire growth cone.

### Discussion of the Parameters

To a large extent, the dynamics of the receptor is governed by the diffusion coefficient 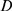. The average value of 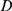 has been measured (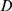 = 0.25 µm^2^.s^−1^) [28] and has been used in all the simulations unless otherwise indicated. The coupling of the receptors dynamics to the MTs is governed by two parameters: the depth 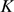 and the extension 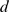 of the potentials 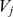 located at the MT ends. The potential introduced in the simulation does not intend to describe interactions at the molecular scale. The temporal resolution of simulations (10 s) implies that the effective potential represents an average interaction over this time scale, not the real molecular interactions. Since interactions are weak, i.e receptors are likely to escape the potential at the MT end in less than 10 s, and receptor present the same diffusion coefficient when interacting with MTs or not [28], the extension has to be the one of the typical region explored by a freely diffusing receptor in 10 s, *i.e.* 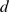
∼1 µm. Furthermore, it means that the shape of 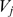 can be taken as a Gaussian in order to account for the diffuse motion of the receptors. When simulations were performed with a time step 1 s, the extension 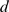 was rescaled accordingly.

Receptors interact but only transiently with MTs, with an average binding time 4.0 s [24], [28]. The depth 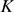 of the potential 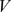 has comparable to thermal fluctuations to permits the diffusive escape of receptors within a few seconds. We have used a value 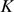 = 3 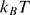, even though a redistribution was still possible for weaker potentials (1 or 2 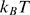). Overall, 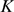 is not a sensitive parameter and simulations performed with 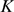 between 2 and 5 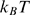 led to similar results (Figure S2).

The regulation of the MT dynamics by the activation field 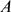 relies on the parameters 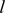, 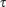 and 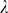. Analogous to 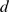, the value of 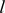 = 1 µm is the typical size of the domain explored by a diffusing receptor between two consecutive simulation times. The MT elongation velocity in GC has been measured, with a typical value of 0.1–1 µm.s^−1^ [59]. Therefore, 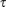 and 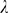 are chosen to keep MT elongation speed in a similar range. Given that the length of a MT remains comparable to 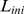 the equilibrium length in the absence of stimulation due to the model geometry, the value 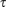 = 10 s and 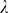 = 0.1 µm.s^−1^ were used in the simulations. Measurements performed on simulation with these set of parameters ensured that MT elongation speed remains in the correct range.

Other important parameters such as the total number 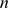 of receptors 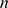 and 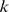 of MTs are not precisely known and different values have thus been numerically tested (see *Results* section). A summary of the parameter values is presented in Table 2.

In order to check that our results do not depend on the time increment (10 s), we performed numerical computations with a time increment of 1 s. The redistribution curve 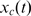 of the receptors in these conditions is very similar to the one obtained with simulations ran at 0.1 Hz (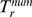 = 10.1 min and 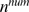 = 3.9). As a result, we used a 10 s increment to reduce computation time, without loss of physical content.

## Supporting Information

Figure S1 Cumulative distribution of receptors for a concentration difference ΔC = 2% (green triangles), 20% (blue circles) and 50% (black squares). Each curve is adjusted by the cumulative distribution in the linear potential W_eff_(x) = −ak_B_Tx respectively with a = 1.01, 1.83 and 2.65.
(0.36 MB TIF)

Figure S2 Redistribution for a well depth of 2 (red) and 5 k_B_T (blue). Curves are normalized to the final amplitude of redistribution.
(0.29 MB TIF)

Figure S3 Comparison (blue dots) of redistribution dynamics with activation defined by comparison to the spatial average (Δx_c_) and by comparison to a fixed value (Δx^′^_c_). The red dashed line is the identity.
(0.69 MB TIF)
