# Supplementary figures and images for: A Mechanism for the Polarity Formation of Chemoreceptors at the Growth Cone Membrane for Gradient Amplification during Directional Sensing
Source: PLoS One. 2010 Feb 22;5(2):e9243. doi: 10.1371/journal.pone.0009243 (PMC2825272; doi:10.1371/journal.pone.0009243)

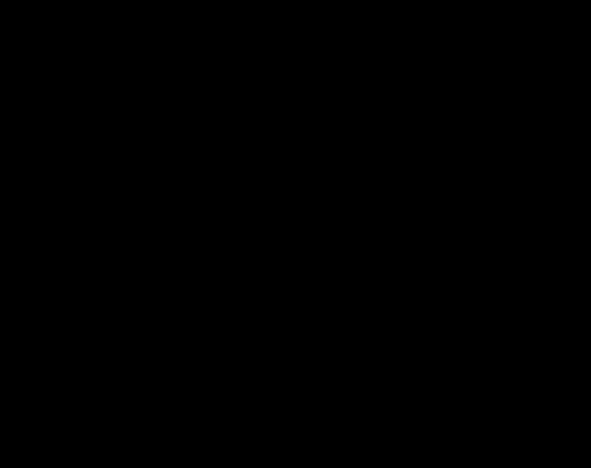

Supplement: Figure S3 — Comparison (blue dots) of redistribution dynamics with activation defined by comparison to the spatial average (Δxc) and by comparison to a fixed value (Δx′ c). The red dashed line is the identity. (0.69 MB TIF) [file pone.0009243.s003.tif]
